# Health-related quality of life in children and adolescents with overweight and obesity: results from the German KIGGS survey

**DOI:** 10.1186/s12889-020-09834-8

**Published:** 2020-11-16

**Authors:** Lara Meixner, Caroline Cohrdes, Anja Schienkiewitz, Gert B. M. Mensink

**Affiliations:** Department of Epidemiology and Health Monitoring, Robert Koch Institute, General-Pape-Str. 62-66, 12101 Berlin, Germany

**Keywords:** Overweight, Obesity, Children, Adolescents, HRQoL, KIDSCREEN-27, KIGGS, Germany, Media consumption, Physical activity

## Abstract

**Background:**

The well-being of persons with overweight and obesity, in particular of children and adolescents, may be impaired. The present study investigates the health-related quality of life (HRQoL) of girls and boys with overweight and obesity living in Germany as compared to those of normal-weight, while taking a selection of relevant determinants of HRQoL into account.

**Methods:**

The sample comprises 1771 children and adolescents aged 11 to 17 years that took part in the cross-sectional German Health Interview and Examination Survey for Children and Adolescents (KIGGS Wave 2, 2014–2017). Sex-and age-specific BMI (kg/m^2^) percentiles were utilized to classify overweight and obesity. HRQoL was measured with the KIDSCREEN-27 questionnaire, which gathers detailed information about the five dimensions physical and psychological well-being, well-being regarding peers (i.e., social acceptance), parents (i.e., autonomy) and within the school environment. Multiple regression analyses were performed with HRQoL dimensions as outcomes to test for differences between children and adolescents with normal-weight vs. those with overweight and vs. those with obesity, separately for girls and boys. In a next step, age, physical activity, media consumption, social support and self-efficacy were considered as potential confounders in the analyses.

**Results:**

18.7% of the children and adolescents under study were affected by overweight and among them 8.0% by obesity. After adjusting for potential confounders, overweight and obesity were associated with lower physical well-being as compared to normal weight in both sexes (boys with overweight: standardized beta [β] = −.14, standard error [SE] = .03, *p* < .001, and obesity: β = −.16, SE = .03, p < .001; girls with overweight: β = −.09, SE = .04, *p* = .011, and obesity: β = −.11, SE = .03, *p* = .003). Results moreover suggest lower levels of psychological (β = −.10, SE = .04, *p* = .002) and parent-related well-being (β = −.08, SE = .04, *p* = .036) of boys with obesity as compared to normal-weight peers.

**Conclusion:**

HRQoL of German children and adolescents with overweight and obesity is impaired according to physical well-being in general, while psychological and parent-related well-being is particularly affected in boys. Public health approaches should therefore promote children and adolescents with overweight and obesity by improving diverse facets of HRQoL as well as relevant associated factors (i.e., media consumption, self-efficacy) in general and in boys in particular.

**Supplementary Information:**

The online version contains supplementary material available at 10.1186/s12889-020-09834-8.

## Background

During the last four decades, the worldwide prevalence of children and adolescents with overweight and obesity has increased considerably [1]. Correspondingly, Germany recorded a substantial increase in the prevalence of child and adolescent obesity in the last decades [2]. Although actual findings suggest that overweight and obesity rates in Germany as well as in and other high income countries reached a plateau, there is still a considerably proportion of 15.4% of German children and adolescents aged between 3 and 17 years that are affected by overweight and among them 5.9% by obesity [1, 3].

Previous findings indicate a relatively high risk of persistence with more than half of the children with overweight and obesity being affected by overweight or obesity also in adolescence and with a five-fold higher risk even in adulthood [4, 5]. This is particularly important considering the individual and social consequences and accompanying symptoms of persistent overweight and obesity. Overweight and obesity among children and adolescents are associated with cardiovascular risk factors (e.g. hypertension, dyslipidemia) as well as metabolic disorders (e.g. glucose intolerance) and can lead to substantial psychosocial problems [6–9]. Moreover, it has been shown that children with overweight and obesity are more likely to be ill, are more often absent from school due to illness and require medical care more frequently than peers with normal weight already during their youth [6]. Children with overweight and obesity also tend to report significantly more musculoskeletal problems than children with normal weight [7]. Frequently, young people with overweight and obesity are affected by weight-related stigmatization, discrimination as well as frequent teasing and bullying by peers causing psychosocial health problems as low self-esteem, body dissatisfaction, eating disorders, depression, anxiety and social isolation [8–10]. Taken together, the adverse consequences of juvenile overweight and obesity on multiple aspects of health and well-being imply that measures beyond health indicators inferred from the classical biomedical model have to be taken into account [11]. As the multidimensional construct of health-related quality of life (HRQoL) covers multiple aspects of health, it allows assessing a comprehensive burden of certain health conditions [12].

HRQoL comprises subjective aspects of quality of life and health [13]. In order to include quality of life aspects within the context of healthcare, HRQoL focuses on life domains that can be influenced by medical or psychosocial interventions [14]. In the course of an increasing prevalence of chronic somatic and mental health conditions in recent years, HRQoL has become a central indicator in public health research as it helps to identify individuals at risk for mental health problems or with hidden morbidity as well as the associated burden and healthcare needs [12, 15–17]. Measuring HRQoL of children and adolescents with overweight and obesity provides important knowledge about how to plan and improve prevention and care in different life domains [12]. In terms of overweight and obesity, measuring HRQoL can be helpful to investigate how a particular treatment form not only affects the weight status, but also the well-being of children and adolescents. Based on this knowledge, the treatment options can be specified and adapted to a certain target group (e.g. intensive long-term treatments for children and adolescents with severe obesity) [18].

Previous findings suggest that children and adolescents with overweight and obesity show significantly lower HRQoL levels as compared to normal-weight peers [17, 19]. However, the affectedness of different HRQoL domains as well as the severity among children and adolescents with overweight and obesity varies depending on age, sex, study population and HRQoL instrument [19, 20]. Results from the first representative German Health Interview and Examination Survey for Children and Adolescents (KiGGS baseline; 2003–2006) substantiate previous indications of adverse effects from overweight and obesity on HRQoL [21]. German children and adolescents with overweight and obesity showed reduced HRQoL levels according to their psychological, physical, peers- and school-related well-being. Additionally, girls with overweight and obesity turned out to be more affected than boys according to their levels of psychological well-being and well-being at school [22].

Explanatory approaches point toward social changes having an impact on HRQoL of young people with weight problems. For instance, comparisons with other people’s self-presentation on social media websites may result in body dissatisfaction and low self-esteem [23]. On the other hand, juvenile overweight and obesity is an extensively discussed problem in media. This may lead to less stigmatization and a greater understanding for the individuals concerned [24]. Furthermore, the fact that the fashion industry is promoting plus-size models may raise especially young girl’s self-esteem [25]. Thus, continuously observing HRQoL of young individuals, while taking relevant determinants of lifestyle and social context into account, is highly important. Evidence suggests that physical activity, media consumption, social support and self-efficacy are likely to influence children’s and adolescent’s HRQoL and are also associated with obesity [20, 26–29]. Thus, with the present analyses we aimed at examining the HRQoL of girls and boys with overweight and obesity in Germany as compared to ones of normal-weight counterparts, in due consideration of a selection of relevant determinants of HRQoL and overweight/obesity. Second, we aimed at identifying the HRQoL dimensions most affected in children and adolescents with overweight and obesity. Since it has been observed that HRQoL levels tend to be lower among girls, particular attention was paid to potential sex differences [30].

## Methods

### Sample and procedure

The present analyses are based on cross-sectional representative data obtained from the second wave of the German Interview and Examination Survey for Children and Adolescents (KIGGS Wave 2, 2014–2017). The concept and design of KiGGS are described in detail elsewhere [31, 32]. In brief, participants were selected randomly from the official population registries in 167 cities and municipalities representative for Germany. In total, 15,023 children and adolescents covering the age range from 0 to 17 years (7538 girls, 7485 boys; response rate 40.1%) participated in KiGGS Wave 2. Of these, 3567 participants (1801 girls, 1766 boys; response rate 41.5%) took part in the examination part. The final sample comprises 1771 children and adolescents (937 girls, 834 boys) aged from 11 to 17 years (M = 13.85, SD = 1.98) who completed the examination part. All participants aged 11–17 years who took part in the examination part reported valid information on HRQoL.

### Measures

#### Health-related quality of life (HRQoL)

HRQoL was measured with the KIDSCREEN-27 questionnaire [33]. With reference to the past week, the KIDSCREEN-27 questionnaire comprises 5 items on physical well-being (e.g. Have you felt fit and well?), 6 items on psychological well-being (e.g. Have you been happy with the way you are?), 7 items on autonomy & parent relation (e.g. Have you had enough time for yourself?), 4 items on social support & peers (e.g. Have you had fun with your friends?) and 4 items on school environment (e.g. Have you got on well at school?) answered on a 5-point rating scale from (0) not at all, to (4) extremely. The KIDSCREEN-27 questionnaire has robust psychometric properties, with high discriminatory power and internal consistency of the subscales [34, 35].

Following the KIDSCREEN scoring instructions, first, the negatively phrased items were recoded; subsequently, sum scores for the respective subscales were calculated; next, raw scores were transformed into Rasch person parameters and finally converted into T-values in order to make their interpretation more applicable [36].

#### Weight status

The anthropometric measurements were conducted by trained staff according to standardized methods. Body height was measured with a portable Harpenden stadiometer (Holtain Ltd., UK) to an accuracy of 0.1 cm, and body weight on a calibrated electronic scale (SECA Ltd., Germany) with a precision of 0.1 kg. Body-Mass Index (BMI) in kg/m^2^ was calculated from body weight divided by the square of body height. Weight status was determined using age- and sex-specific cut-offs for underweight (<10th percentile), normal weight (≥10th percentile to ≤90th percentile), overweight (>90th percentile) and obesity (>97th percentile) based on the reference percentiles according to the national reference system [37].

#### Determinants

Age, sex, socioeconomic status (SES), physical activity, media consumption, social support and self-efficacy were considered as potential confounders. SES was reflected by an index including the level of parental education, occupational status and the household income. The index was categorized into low, medium and high SES [38].

The amount of physical activity was assessed via the question: “On how many days of a normal week are you physically active for at least 60 minutes on a single day?” Within KIGGS Wave 2, physical activity on more than 6 days per week was defined as sufficiently physically active (achievement of the WHO recommendations of physical activity), between 2 and 6 days as medium physical activity and less than two days a week as low physical activity [39].

Regarding media consumption, children and adolescents were asked how much time they spend on average per day watching TV/video as well as using the computer, internet, game console and social media. In order to determine the average daily media consumption, an additive overall daily average index was calculated. Based on the distribution of the sample, participants were defined as moderate users if they spend 2.5 h or less, as frequent users if they spend more than 2.5 h up to 4.5 h and as extensive users if they spend more than 4.5 h per day with electronic media.

Social support was measured via the self-report short version of the Social Support Survey (SSS-short). By means of a 5-point rating scale from 0 to 4 (0 = none of the time to 4 = all of the time), the 8 items the questionnaire assesses how often the children or adolescents receive different types of social support (e.g. “How often is the following type of support available for you if you need it? ”) [40].

The General Self-Efficacy Scale was used to assess self-reported self-efficacy with 10 items (e.g, “It is easy for me to stick to my aims and accomplish my goals.”) answered on a 4-point rating scale from 0 to 3 (0 = not at all true to 3 = exactly true) [41].

To report sample characteristics (Table 1), participants were categorized into groups of low (< 26%), medium (> 25 and < 76%) and high (> 75%) social support and self-efficacy based on the distribution of the sample. For regression analyses (Tables 2 and 3), we used the respective sum scores.

**Table 1:** Sample Characteristics in Total and Separately for Girls and Boys

	Total (***n*** = 1770)		Girls 52.9% (***n*** = 936)		Boys 47.1% (***n*** = 834)	
	n	% [95% CI]	n	% [95% CI]	n	% [95% CI]
**Age group**						
11–13 years	815	40.8 [37.9;43.9]	410	41.0 [37.0;45.3]	405	40.6 [36.4;45.0]
14–17 years	955	59.2 [56.1;62.1]	526	59.0 [54.7;63.0]	429	59.4 [55.0;63.6]
**Weight status**						
Underweight	154	8.4 [6.1;11.2]	73	7.5 [4.7;13.2]	81	9.1 [6.0;13.8]
Normal weight	1322	72.9 [70.0;75.7]	714	74.8 [70.7;78.4]	608	71.2 [66.7;75.3]
Overweight	171	10.7 [8.79;13.0]	94	10.6 [8.1;13.7]	77	10.9 [8.1;14.5]
Obesity	123	8.0 [6.4;10.0]	55	10.3 [5.1;10.0]	68	8.7 [6.4;11.8]
**Socioeconomic status**						
Low	260	22.6 [19.7;25.8]	142	22.4 [18.9;27.6]	118	22.3 [18.3;26.8]
Moderate	1086	60.0 [56.8;63.1]	579	60.6 [56.0;64.8]	507	59.6 [54.9;64.0]
High	372	17.4 [15.3;19.7]	187	16.5 [15.2;21.6]	185	18.2 [15.2;21.6]
**Physical activity**						
Low	212	14.4 [12.2;16.9]	157	20.1 [17.2;24.7]	55	8.4 [6.0;11.7]
Medium	1224	69.2 [66.1;72.1]	641	68.5 [65.4;74.0]	583	69.9 [64.1;72.4]
Sufficient	271	16.4 [14.2;18.9]	102	10.8 [08.5;13.8]	169	21.6 [18.0;25.7]
**Media consumption**						
Moderate	407	22.0 [19.7;24.7]	220	24.3 [20.8;28.2]	187	20.1 [16.9;23.7]
Frequent	403	21.6 [19.3;24.2]	216	22.5 [19.3;26.1]	187	20.8 [17.5;24.6]
Extensive	960	56.2 [53.2;59.3]	501	53.2 [48.9;57.4]	460	59.2 [54.8;63.4]
**Social support**						
Low	488	29.6 [26.7;32.6]	199	21.2 [18.0;24.9]	289	37.5 [33.1;42.1]
Moderate	776	44.9 [41.7;48.0]	415	46.5 [42.2;50.9]	362	43.3 [38.9;47.8]
High	436	25.6 [23.0;28.4]	287	32.3 [28.4;36.5]	149	19.2 [15.9;22.9]
**Self-efficacy**						
Low	511	30.4 [27.5;33.4]	315	35.8 [31.7;40.1]	196	25.2 [21.3;29.5]
Moderate	745	43.5 [40.5;46.7]	374	40.6 [36.3;45.0]	371	46.5 [41.9;51.1]
High	418	26.0 [23.3;28.9]	204	23.6 [20.0;27.4]	214	28.3 [24.2;32.8]
**HRQoL**^**1**^						
Physical well-being		48.5 [47.9;49,1]		46.9 [46.0;47.7]		50.1 [49.2;50.9]
Psychological well-being		50.7 [50.1;51.3]		49.2 [48.3;50.1]		52.1 [51.2;52.9]
Autonomy & Parent relation		54.0 [53.5;54.6]		53.4 [52.7;54.2]		54.6 [53.8;55.4]
Social support & peers		51.4 [50.8;52.0]		51.9 [51.1;52.6]		51.0 [50.1;51.8]
School environment		51.1 [50.5;51.6]		51.1 [50.4;51.8]		51.0 [50.2;51.9]

*Notes.* N = unweighted number and % = Weighted proportions.^1^ T-values

**Table 2:** Predicting Health-Related Quality of Life (Physical and Psychological Well-being, Autonomy and Parent Relation, Social Support and Peers, School Environment) by Children’s and Adolescent’s Weight Status separately for Girls (*n* = 936) and Boys (*n* = 834), While Controlling for Age and the Socioeconomic Status

	Physical well-being		Psychological well-being		Autonomy & parent relation		Social support & peers		School environment	
	*B (SE)*	*p*	*B (SE)*	*p*	*B (SE)*	*p*	*B (SE)*	*p*	*B (SE)*	*p*
**Girls**										
Age	**−1.26 (0.22)**	**<.001**	**− 1.17 (0.22)**	**<.001**	**−0.78 (0.20)**	**<.001**	**− 0.44 (0.20)**	**.003**	**− 0.81 (0.18)**	**<.001**
Low SES	Ref.		Ref.		Ref.		Ref.		Ref.	
Moderate SES	0.83 (1.19)	.484	−2.06 (1.48)	.164	−0.04 (1.00)	.965	− 0.68 (1.02)	.508	−1.12 (1.07)	.291
High SES	1.99 (1.36)	.145	−1.23 (1.59)	.440	2.06 (1.36)	.134	−1.23 (1.07)	.252	0.79 (1.20)	.510
Normal weight	Ref.		Ref.		Ref.		Ref.		Ref.	
Underweight	0.21 (1.30)	.870	−0.19 (1.08)	.862	−1.32 (1.26)	.279	0.42 (1.48)	.780	−0.11 (1.13)	.924
Overweight	**−2.91 (1.31)**	**.024**	−0.77 (1.38)	.580	−1.30 (1.14)	.256	−0.77 (1.14)	.500	−0.86 (1.19)	.467
Obesity	**−6.56 (2.06)**	**.001**	−4.95 (2.54)	.052	−1.90 (2.22)	.393	−3.80 (2.15)	.077	0.04 (2.23)	.986
*R*^2^	.12		.08		.05		.02		.04	
**Boys**										
Age	**−0.55 (0.22)**	**.012**	**−0.54 (0.20)**	**.007**	0.12 (0.21)	.571	−0.17 (0.21)	.432	−0.31 (0.22)	.168
Low SES	Ref.		Ref.		Ref.		Ref.		Ref.	
Moderate SES	0.79 (1.18)	.499	1.83 (1.13)	.107	1.84 (1.14)	.106	2.44 (1.30)	.061	2.41 (1.45)	.095
High SES	0.95 (1.29)	.459	1.75 (1.28)	.169	2.24 (1.33)	.093	**2.77 (1.40)**	**.049**	**4.08 (1.57)**	**.010**
Normal weight	Ref.		Ref.		Ref.		Ref.		Ref.	
Underweight	−0.60 (1.15)	.598	1.37 (1.40)	.328	0.79 (1.32)	.545	0.81 (1.67)	.625	1.30 (0.94)	.170
Overweight	**−4.73 (1.28)**	**<.001**	−1.38 (1.51)	.363	−1.94 (1.49)	.175	−2.27 (1.25)	.068	−0.98 (1.75)	.587
Obesity	**−6.27 (1.53)**	**<.001**	**−3.48 (1.15)**	**.003**	−2.35 (1.21)	.052	−2.66 (1.80)	.141	−1.38 (1.83)	.453
*R*^2^	.07		.04		.08		.04		.04	

*Notes. B* Unstandardized beta, *SE* Standard error, *SES* Socioeconomic status. Significant results with *p* < .05 are highlighted in boldface

**Table 3:** Predicting Health-Related Quality of Life Domains (Physical and Psychological Well-Being, Autonomy and Parent Relation) by Children’s and Adolescent’s Weight Status separately for Girls (*n* = 936) and Boys (*n* = 834), While Controlling for Relevant Health Determinants

	Girls			Boys		
	***B (SE)***	β	***p***	***B (SE)***	β	***p***
**Physical well-being**						
Age	**−0.64 (0.21)**	**−.13**	**.003**	−0.12 (0.19)	−.03	.544
Low SES	Ref.			Ref.		
Moderate SES	1.03 (1.01)	.05	.311	**−2.27 (0.93)**	**−.12**	**.015**
High SES	1.00 (1.20)	.04	.407	**−2.53 (1.08)**	**−.11**	**.020**
**Weight status**						
Normal weight	Ref.			Ref.		
Underweight	0.14 (0.92)	<.01	.882	0.25 (1.04)	.01	.813
Overweight	**−2.81 (1.10)**	**−.09**	**.011**	**−4.35 (1.05)**	**−.14**	**<.001**
Obesity	**−4.55 (1.54)**	**−.11**	**.003**	**−5.77 (1.17)**	**−.16**	**<.001**
**Determinants**						
Media consumption	**−0.52 (0.11)**	**−.19**	**<.001**	**−0.57 (0.12)**	**−.20**	**<.001**
Physical activity	**1.42 (0.22)**	**.29**	**<.001**	**1.63 (0.19)**	**.35**	**<.001**
Social support	0.05 (0.03)	.08	.129	**0.09 (0.02)**	**.17**	**<.001**
Self-efficacy	**0.12 (0.03)**	**.19**	**<.001**	**0.10 (0.02)**	**.16**	**<.001**
*R*^2^	.34			.34		
**Psychological well-being**						
Age	**−0.75 (0.19)**	**−.15**	**<.001**	−0.37 (0.20)	−.08	.070
Low SES	Ref.			Ref.		
Moderate SES	−1.79 (1.24)	−.09	.170	−0.78 (1.06)	−.04	.464
High SES	−1.97 (1.43)	−.08	.170	−1.00 (1.22)	−.04	.412
**Weight status**						
Normal weight	Ref.			Ref.		
Underweight	−0.25 (1.02)	−.01	.806	1.63 (1.14)	.05	.152
Overweight	−0.38 (1.18)	−.01	.748	0.16 (1.25)	.01	.897
Obesity	−2.49 (1.89)	−.06	.188	**−3.52 (1.14)**	**−.10**	**.002**
**Determinants**						
Media consumption	**−0.26 (0.11)**	**−.10**	**.015**	−0.19 (0.12)	−.07	.114
Physical activity	0.27 (0.17)	.05	.131	**0.59 (0.21)**	**.13**	**.005**
Social support	**0.16 (0.02)**	**.24**	**<.001**	**0.10 (0.03)**	**.19**	**.001**
Self-efficacy	**0.22 (0.03)**	**.37**	**<.001**	**0.18 (0.03)**	**.31**	**<.001**
*R*^2^	.36			.26		
**Autonomy and parent relation**						
Age	**−0.72 (0.20)**	**−.16**	**<.001**	0.19 (0.21)	.04	.382
Low SES	Ref.			Ref.		
Moderate SES	−0.06 (1.00)	<−.01	.952	0.55 (1.08)	.03	.611
High SES	1.16 (1.38)	.05	.401	0.38 (1.24)	.02	.763
**Weight status**						
Normal weight	Ref.			Ref.		
Underweight	−1.04 (1.24)	−.03	.403	1.19 (1.15)	.04	.304
Overweight	−1.08 (1.14)	−.04	.341	−0.05 (1.36)	<−.01	.971
Obesity	0.32 (2.26)	.01	.889	**−2.66 (1.27)**	**−.08**	**.036**
**Determinants**						
Media consumption	−0.10 (0.11)	−.04	.370	−0.12 (0.13)	−.05	.327
Physical activity	0.03 (0.18)	.01	.886	−0.07 (0.22)	−.02	.760
Social support	**0.14 (0.03)**	**.23**	**<.001**	**0.11 (0.03)**	**.21**	**<.001**
Self-efficacy	**0.11 (0.03)**	**.21**	**<.001**	**0.12 (0.03)**	**.21**	**<.001**
*R*^2^	.19			.15		

*Notes*. *B* Unstandardized beta, *SE* Standard error, *β* Standardized beta, *SES* Socioeconomic status. Determinants were entered as continuous variables. Significant results with *p* < .05 are highlighted in boldface

### Statistical analyses

The analyses were conducted with a weighting factor. This factor was applied to correct deviations of the sample from the German population structure with regard to age, gender, federal state and the parents’ level of education during the time of the survey [31]. Furthermore, we took the clustered design of the participants within the 167 sample points across Germany into account by performing all analyses using survey procedures for complex samples in SAS 9.4 (SAS Institute, Cary NC, USA) [32]. The survey variance estimator taking clustering and weighting into account is based on Taylor linearization and is very similar to the robust variance estimator (sandwich estimator) used in GEE (generalized estimating equations) models.

#### Sample characteristics

First, we computed descriptive sample statistics for groups stratified by the determinants of age, SES, BMI, physical activity, media consumption, social support, self-efficacy and HRQoL in total and separated by sex.

#### Regression analyses

Next, we performed multiple regression analyses to investigate differences in the five HRQoL dimensions of physical well-being, psychological well-being, autonomy and parent relation, social support and peers and school environment between normal-weight children and adolescents and those with overweight and obesity while controlling for the participant’s age and SES (Model 1). Next, in Model 2 we rerun the analyses but additionally adjusted for relevant health determinants as potential confounders (physical activity, media consumption, social support and self-efficacy). Overweight and obesity were included in the model as independent variables and “normal weight” as the reference category. Except from weight status and SES, all other variables (media consumption, physical activity, social support and self-efficacy) were included into the regression models as continuous variables. Both the adjusted and unadjusted models are conducted separately for girls and boys in order to examine sex differences. Differences in HRQoL between the groups of obesity, overweight and normal weight were interpreted as statistically significant if the calculated *p*-value was smaller than 0.05. To compare the relative effects of the weight status and the potential cofounders on the five HRQoL dimensions, standardized regression coefficients were calculated for the fully adjusted model (Model 2).

#### Post-hoc power analysis

Results from post-hoc power analyses using G*Power [42] suggest that the present sample size of *n* = 730 boys respectively *n* = 807 girls was sufficient for the detection of moderate effects (f = 0.15), within a multiple regression design with six predictors and an error probability of α = 0.05, at the power level of 1.00.

## Results

### Sample characteristics

Sample characteristics of the study participants are presented in Table 1 by gender, 52.9% (*n* = 936) were female and 47.1% (*n* = 834) were male. 40.8% of the participants were aged between 11 and 13 years, 59.2% were 14 to 17 years old. The majority (72.9%) had normal weight, while around 11% were affected by overweight and 8% by obesity. In line with the definition by quintiles, nearly two thirds were categorized as middle SES, 22.6% were assigned to have a low SES and 17.4% to have a high SES [38]. Most of the children and adolescents reported medium physical activity (69.2%). Compared to boys, the proportion of girls reporting low physical activity was about twice as high (10.8% vs. 21.6%). Notably, more than half of the participants were classified as extensive media users (56.2%). The distribution of media consumption was similar among girls and boys. Approximately one quarter of the children and adolescents (25.6%) received high levels of social support and 29.6% low levels of social support. The amount of boys with high levels of social support was almost 13% lower and the proportion of boys with low social support was about 16% higher compared to girls. More than one third (43.5%) was assigned to have low self-efficacy and 26.0% to have high self-efficacy. The proportion of girls reporting low self-efficacy was about 10% higher compared to boys. In general, German child and adolescent HRQoL dimensions correspond to European norm values or are even better (centered around T-value .50). One exception is represented by physical well-being which falls slightly below the European norm with a mean value of 48.5.

### HRQoL of girls and boys with differing weight status

Table 2 shows the results of the five regression models testing associations between the weight status and the five HRQoL dimensions, separately for girls and boys (Model 1). Both girls and boys affected by overweight and obesity had significantly poorer physical well-being than those with normal weight. Results further indicate that boys with obesity had lower psychological well-being while the association was not significant for girls (Table 2).

### Determinants of HRQoL in girls and boys

Results from Model 2 adjusted for relevant health determinants (media consumption, physical activity, social support and self-efficacy) replicate findings from Model 1: girls and boys with overweight or obesity have reduced physical well-being levels as compared to those with normal weight; boys with obesity have reduced psychological well-being (Table 3). In addition, we found a significant negative association between well-being regarding autonomy and parents and obesity in boys.

Besides overweight and obesity, higher levels of media consumption were associated with lower physical well-being in general and with lower psychological well-being in particular in girls. High levels of physical activity, self-efficacy and social support, on the contrary, were related to better physical, psychological and parent-related well-being. Overall, physical activity had the comparatively strongest positive effect on physical well-being in both girls and boys (standardized beta of .29 respectively .35) while self-efficacy had the strongest effects on psychological (for girls .37 and for boys.31) and parent-related well-being (for girls and boys .21). Associations between obesity and well-being with peers and in school were non-significant in girls and only marginally significant in boys. Detailed results can be obtained from Table S1 of the Supplementary material.

## Discussion

The primary aim of the present analyses was to examine HRQoL levels of children and adolescents with overweight and obesity in comparison to normal-weight peers based on actual data from the KiGGS study. A further aim was to identify the dimensions of HRQoL mostly affected by overweight and obesity, separately for girls and boys. Moreover, we took several determinants of HRQoL into account, to adjust for potential confounding of associations between overweight/obesity and HRQoL.

### HRQoL in children and adolescents with differing weight status

Girls and boys with overweight and obesity living in Germany showed reduced physical well-being. This result is in line with results from the KiGGS baseline study [22] as well as with other studies examining the relationship between the weight status and HRQoL [43]. It is be possible that certain co-morbidities associated with juvenile overweight and obesity (e.g. musculoskeletal problems) lead to lower levels of physical HRQoL. However, our cross-sectional study design does not allow drawing any conclusion about whether certain co-morbidity associated with overweight and obesity causes lower levels of physical HRQoL or if already impaired physical HRQoL causes individuals to gain weight.

The observed differences in psychological well-being were similar for girls and boys with obesity as compared to normal-weight peers, but only the association for boys with obesity was significant. As far as we know, this finding is novel and surprising considering that girls have been discussed to be more vulnerable towards psychological problems related to overweight/obesity [44]. Two systematic reviews, showed conflicting results regarding differences in psychological well-being related to the weight status [20, 43], most probably related to differences in population and severity of obesity. Many of the studies that observed lower levels of psychological well-being among girls and boys affected by obesity used data from clinical samples or from children and adolescents with severe obesity. Substantial impairments in psychological well-being among girls and boys in these studies may result from the fact that treatment was sought because of profoundly lowered HRQoL levels. Moreover, children and adolescents with severe obesity may suffer from associated conditions, limitations or diseases more seriously than children and adolescents with overweight or milder forms of obesity. Since KIGGS is a population-based survey, the observed differences may be smaller. Nevertheless, there are also population-based studies that observed lower levels of psychological well-being in both girls and boys with overweight and obesity [45] or in girls, only [22]. Another explanation refers to the fact that girls may no longer exclusively compare themselves with thin women presented in media, but also with popular average or plus-size models. The so-called “body positivity movement” especially addresses girls and aims to end weight-related stigmatization in media [46]. However, it seems that little consideration has been given to the fact that boys may also experience the pressure to look attractive when influenced by social media. In 2014, de Vries and colleagues observed that frequent social media use is a predictor for an increased investment in appearance and relationships between appearance pressures, investment in appearance and appearance-changing activities were similarly evident in both girls and boys [47]. These findings suggest that, regardless of sex, body image may be an important issue for children and adolescents and may substantially influence their psychological well-being and that boys might need more public health attention.

In contrast to several previous findings, we found no associations between the weight status and well-being regarding peer relations and a significant association with parent-related well-being became evident in boys only. Based on previous findings we expected particularly negative effects from overweight/obesity on social well-being. Within their systematic review, Tsiros et al. concluded that similar to physical well-being, social functioning is the dimension most seriously impaired in children and adolescents with overweight and obesity [43]. Notably, they only incorporated studies using the Pediatric Quality of Life Inventory (PedsQL) questionnaire to assess HRQoL in their review. Comparing the PedsQL and the KIDSCREEN-27 questionnaire, it becomes obvious that the questions on social well-being differ, in particular PedsQL is more focused on bullying [48]. Since children and adolescents with overweight and obesity are known to be affected by teasing more frequently than normal weight peers, this could be the reason for more frequent observations of impairments in social well-being within higher BMI classes when assessing HRQoL with the PedsQL [8]. Differences in the content of varying HRQoL questionnaires may also have contributed to the inconclusive findings regarding parent-related well-being and HRQoL: while the KIDSCREEN-27 instrument investigates how independent children and adolescents feel from their parents, this aspect is not measured via other HRQoL instruments. Thus, the present study yields new evidence suggesting that obese boys feel more dependent on their parents than those with normal weight. Future studies might want to differentiate and validate the relevance of single HRQoL items from different instruments for the group of obese children or adolescents in more detail. The question why this pertains only to boys but not to girls remains unclear and awaits further exploration.

Regarding well-being in the school environment, girls and boys affected by overweight and obesity did not report lower levels of HRQoL than normal-weight counterparts. In line with this finding, Tsiros et al. (2009) and Buttitta et al. (2014) came based on their systematic reviews to the conclusion that well-being in school is not associated with overweight and obesity among children and adolescents [20, 43]. Results further imply that experiences of discrimination and bullying of children and adolescents with overweight or/and obesity take place outside of the school context, which awaits further investigation.

### The role of relevant determinants for HRQoL

Independent of the weight status, older age was associated with reduced HRQoL levels among girls concerning all the HRQoL dimensions whereas this was true for physical and psychological well-being in boys, only. This finding is supported by similar patterns of age and sex differences from other studies [49]. Adolescent girls seem to be more susceptible to reduced HRQoL during puberty because they generally showed more problems with transitions regarding social and sexual roles than adolescent boys [50].

Furthermore, low physical activity and high media consumption were associated with lower HRQoL and physical well-being in particular. It has already been shown that young people reporting low physical activity are more often affected by physical and mental health problems than children and adolescents who regularly exercise [51]. Regular physical activity moreover seems to be associated with a more positive self-image [52]. Correspondingly, the present results revealed associations between high levels of self-efficacy as well as social support with good HRQoL according to all five dimensions [26]. Thus, the present findings underpin previous indications of positive social relationships supporting a variety of well-being domains [53]. Moreover, high self-efficacy has been discussed as an individual resource for HRQoL by showing confidence in personal coping abilities across a wide range of demanding situations in various contexts [54].

### Strengths and limitations

A major strength of the KiGGS cross-sectional study is the sampling design, conduction and weighting including many efforts to improve the participation rate. The results can be generalized for the young German population and thereby add value to a predominant research bias toward clinical samples and young people already seeking treatment. The representativeness of the cross-sectional KIGGS data was ensured by several complex measures as described in Hoffmann et al. (2018) [31]. First, an adequate and well-established method for drawing the samples was used. Second, numerous measures such as incentives, multiple forms and repeated invitations including telephone calls and the provision of questionnaires in various languages were used to reach several population groups and achieve high levels of participation and the most possible unbiased sample composition. Third, response rate analyses were used to examine deviations of the study sample from the German population structure regarding central demographic and health characteristics (i.e., age, sex, federal state, foreigner status and household education level). Last, a weighting was calculated and used to adjust for response bias, where necessary. Nevertheless, bias resulting from selective non-participation cannot be fully excluded [31]. The study includes a sufficient spectrum of information, allowing for multiple adjustments within the models. Hence, results contribute to a better understanding of potential confounders and offer prevention approaches in addition to relations between overweight/obesity and HRQoL. Body height and weight were measured by trained staff according to standardized methods instead of using the information provided by the children and parents themselves. An additional strength of KIGGS is the utilization of an internationally developed and standardized HRQoL measurement tool, which has been shown to have good psychometric properties. Moreover, the KIDSCREEN-27 questionnaire was translated into German language by using a standardized translation methodology according to international cross-cultural translation guidelines [55]. Therefore, the results of the present study are internationally comparable. Nevertheless, a few limitations have to be considered. KiGGS is a cross-sectional study, which does not allow any conclusions about the causal direction of the observed effects [31]. Consequently, we are not able draw conclusions about whether already low levels of physical well-being might have led to overweight or obesity by a reluctant engagement in physical activities. However, a lack of physical activity associated with low physical well-being represents a risk for the persistence of overweight or obesity in adulthood.

## Conclusions

Taken together, the HRQoL of German children and adolescents with overweight or obesity is affected according to physical well-being in general and concerning psychological as well as parent-related well-being particularly in boys. Although cause and consequence are not yet well understood, public health interventions should promote weight loss in children and adolescents with overweight and obesity in general. Taking the present and other results into account, weight loss may ultimately lead to higher levels of physical and psychological HRQoL. Additionally, public health approaches should continue and reinforce to pay attention to young people with overweight and obesity in improving their physical well-being. Present findings indicate that interventions focusing on regular physical activity and improving self-efficacy might be most promising, followed by the reduction of the time spent with consuming different media and the facilitation of social support. By addressing such maladaptive behaviors (i.e., extensive media consumption) or lack of personal resources (i.e. low self-efficacy) associated, the negative effects of overweight and obesity among children and adolescents may be attenuated. Results moreover suggest that well-established public health measures should be questioned and adjusted to the particular objective of targeting adolescent boys. In order to provide more precise recommendations regarding public health approaches targeting boys, further research is needed helping to identify specific factors associated with impaired physical and psychological well-being among children and adolescents with overweight and obesity.

## Supplementary Information

**Additional file 1 Table S1.** Predicting Health-Related Quality of Life Domains (Social Support & Peers, School Environment) by Children’s and Adolescent’s Weight Status Separately for Girls (n = 936) and Boys (n = 834), While Controlling for Relevant Health Determinants.

## Data Availability

The datasets generated and/or analyzed during the current study are not publicly available because informed consent from study participants did not cover public deposition of data. A scientific use file with almost all variables of KiGGS Wave 2 is available on request from the Health Monitoring’ Research Data Centre at the Robert Koch Institute in Berlin, Germany (e-mail: fdz@rki.de).

## Abbreviations

BMI: Body Mass Index
CI: Confidence interval
HRQoL: Health-related quality of life
KIGGS: German Health Interview and Examination Survey for Children and Adolescents
SE: Standard error
SES: Socioeconomic status
WHO: World Health Organization
