# Supplementary material for: Health-related quality of life in children and adolescents with overweight and obesity: results from the German KIGGS survey
Source: BMC Public Health. 2020 Nov 16;20:1722. doi: 10.1186/s12889-020-09834-8 (PMC7670618; doi:10.1186/s12889-020-09834-8)
Supplement: Supplementary file 1 — Additional file 1 Table S1. Predicting Health-Related Quality of Life Domains (Social Support & Peers, School Environment) by Children’s and Adolescent’s Weight Status Separately for Girls (n = 936) and Boys (n = 834), While Controlling for Relevant Health Determinants. [file 12889_2020_9834_MOESM1_ESM.docx]

Supplementary Material

Table S1

*Predicting Health-Related Quality of Life Domains (Social Support & Peers, School Environment) by Children’s and Adolescent’s Weight Status Separately for Girls (n=936) and Boys (n=834), While Controlling for Relevant Health Determinants*

|  | **Girls** | | | **Boys** | | |
| --- | --- | --- | --- | --- | --- | --- |
|  | ***B (SE)*** | **β** | ***p*** | ***B (SE)*** | **β** | ***p*** |
| **Social Support & Peers** | | | | | | |
| Age | -0.39  (0.22) | -.08 | .077 | -0.03 (0.23) | -.01 | .910 |
| Low SES | Ref |  |  | Ref |  |  |
| Moderate SES | -0.60  (0.96) | -.03 | .531 | 0.44  (1.38) | .23 | .747 |
| High SES | -1.87  (1.04) | -.08 | .072 | 0.37 (1.44) | .02 | .799 |
| **Weight status** |  |  |  |  |  |  |
| Normal weight | Ref |  |  | Ref |  |  |
| Underweight | 1.10  (1.28) | .03 | .387 | 1.26 (1.49) | .04 | .398 |
| Overweight | -0.70  (1.11) | -.02 | .532 | 0.33  (1.28) | .01 | .795 |
| Obesity | -2.01  (1.94) | -.05 | .300 | -3.17  (1.91) | -.09 | .099 |
| **Determinants** |  |  |  |  |  |  |
| Media consumption | -0.06  (0.13) | -.02 | .650 | -0.49 (0.15) | -.02 | .742 |
| Physical activity | 0.18  (0.19) | .04 | .340 | 0.16 (0.22) | .03 | 0.482 |
| Social support | **0.21**  **(0.04)** | **.33** | **<.001** | **0.18 (0.03)** | **.32** | **<.001** |
| Self-efficacy | **0.08**  **(0.03)** | **.15** | **0.002** | **0.11 (0.03)** | **.18** | **.001** |
| *R*^2^ | .21 | | | .21 | | |
| **School Environment** | | | | | | |
| Age | **-0.46**  **(0.17)** | **-.11** | **.006** | -0.03 (0.20) | -.01 | .869 |
| Low SES | Ref |  |  | Ref |  |  |
| Moderate SES | -1.22  (0.94) | -.07 | .195 | -0.72 (0.96) | -.04 | .453 |
| High SES | -0.80  (1.07) | -.04 | .457 | -0.09 (1.17) | <-.01 | .941 |
| **Weight status** |  |  |  |  |  |  |
| Normal weight | Ref. |  |  | Ref. |  |  |
| Underweight | -0.10  (1.08) | <-.01 | .924 | 1.76 (1.04) | .06 | .092 |
| Overweight | -0.29  (1.04) | -.01 | .783 | 0.14 (1.55) | <.01 | .931 |
| Obesity | 2.09  (1.78) | .06 | .242 | -2.38 (1.41) | -.07 | .091 |
| **Determinants** |  |  |  |  |  |  |
| Media consumption | **-0.57**  **(0.12)** | **-.25** | **<.001** | **-0.40**  **(0.12)** | **-.16** | **.001** |
| Physical activity | -0.13  (0.19) | -.03 | .503 | 0.13  (0.20) | .03 | .449 |
| Social support | **0.07**  **(0.03)** | **.11** | **.021** | 0.05 (0.03) | .10 | .056 |
| Self-efficacy | **0.17**  **(0.02)** | **.32** | **<.001** | **0.18 (0.03)** | **.33** | **<.001** |
| *R*^2^ | .26 | | | .21 | | |

*Notes*. B=Unstandardized beta, SE=Standard error, β=Standardized beta, SES=Socioeconomic status, SES=Socioeconomic status. Determinants were entered as continuous variables. Significant results with *p*<.05 are highlighted in **boldface**.
